# Neutrophil and neutrophil extracellular trap involvement in neutrophilic asthma: A review

**DOI:** 10.1097/MD.0000000000039342

**Published:** 2024-08-23

**Authors:** Yuemu Li, Tianyi Yang, Baihua Jiang

**Affiliations:** a Institutes of Integrative Medicine, Heilongjiang Provincial Hospital of Traditional Chinese Medicine, Heilongjiang, China.

**Keywords:** chemoattractant, neutrophil extracellular traps, neutrophil recruitment, neutrophilic asthma

## Abstract

Asthma is a highly prevalent chronic inflammatory disease characterized by variable airflow obstruction and airway hyperresponsiveness. Neutrophilic asthma (NA) is classified as “type 2 low” asthma, defined as 65% or more neutrophils in the total cell count. There is no clear consensus on the pathogenesis of NA, and the accumulation of neutrophils and release of neutrophil extracellular traps (NETs) may be responsible for its development. A NET is a large extracellular meshwork comprising cell membrane and granule proteins. It is a powerful antimicrobial defence system that traps, neutralizes, and kills bacteria, fungi, viruses, and parasites and prevents the spread of microorganisms. However, dysregulation of NETs may lead to chronic airway inflammation, is associated with worsening of asthma, and has been the subject of major research advances in chronic lung diseases in recent years. NA is insensitive to steroids, and there is a need to find effective biomarkers as targets for the treatment of NA to replace steroids. This review analyses the mechanisms of action between asthmatic neutrophil recruitment and NET formation and their impact on NA development. It also discusses their possible therapeutic significance in NA, summarizing the advances made in NA agents and providing strategies for the treatment of NA, provide a theoretical basis for the development of new therapeutic drugs, thereby improving the level of diagnosis and treatment, and promoting the research progress in the field of asthma.

## 1. Introduction

Asthma is a heterogeneous disease that manifests as a chronic inflammatory response in the airways and afflicts more than 300 million people worldwide.^[1]^ It is characterized by variable airflow obstruction and airway hyperresponsiveness (AHR). Environmental factors such as allergens are major triggers of the narrow airway response in asthma. The physiology of asthma is characterized by bronchial hyperresponsiveness, a tendency of airway smooth muscle to contract when stimulated by allergens. This leads to acute airway narrowing, which is relieved by bronchodilator therapy. However, some patients with asthma that show poor disease control require high doses of inhaled steroids, a second medication, and systemic corticosteroids to stabilize the disease, and reductions in dosage can exacerbate symptoms. Some patients with more severe symptoms cannot control their condition even at the highest medication dose. Asthma that is difficult to control medically is called severe asthma,^[2]^ which accounts for 5% to 10% of the patients with asthma and consumes more than 60% of the total disease costs,^[3]^ representing a global treatment challenge. In recent years there have been considerable advances in the study of the underlying molecular mechanisms or endotypes in asthma. As a result, asthma has been separated into 2 population groups.^[4]^ The first group is “type 2 high” asthma, which includes allergic and eosinophilic asthma characterized by secretion of high levels of interleukin (IL)-4, IL-5, and IL-13. This promotes an increase in the number of eosinophils, periplasmic serum proteins, and total IgE, leading to an increase in airway mucus and bronchial hyperresponsiveness. The other group, “type 2 low” asthma, includes neutrophilic asthma (NA) and obesity asthma, usually without T2 biomarkers or elevated eosinophils.

Targeting neutrophils and neutrophil extracellular traps (NETs) has been highly effective in the treatment of NA in recent years. Neutrophils are the most abundant circulating leukocytes with extensive heterogeneity^[5]^, accounting for approximately 50% to 70% of all leukocytes^[6]^ and representing early response cells at sites of inflammation. They are the first line of defence against bacteria and fungi and have potent antimicrobial properties, killing pathogens through degranulation phagocytosis, producing reactive oxygen species (ROS), and releasing NETs.^[7]^ However, inappropriate or dysregulated neutrophil activation can lead to their excessive recruitment and infiltration, damaging the host and inducing autoimmune and inflammatory diseases.^[8]^ Therefore, we need to focus on the triggering mechanism of neutrophil recruitment in NA.

A NET is a large extracellular meshwork of membrane and granule proteins assembled on a scaffold of depolymerized chromatin and coated with nuclear proteins (e.g., histones), granule proteins (e.g., neutrophil elastase [NE] and myeloperoxidase [MPO]), and cytoplasmic proteins (e.g., S100 calcium-binding proteins A8, A9, and A12, as well as actin and α-actinin).^[9]^ NETs (NET formation, also known as NETosis) capture, neutralize, and kill microorganisms to prevent the spread of infection and thus play an important role in pathogen defence.^[10]^ However, NETs can also lead to toxic effects in the host, and dysregulation of NETs can exacerbate inflammatory responses and damage tissues.^[11]^ Therefore, understanding how NETs are dysregulated and play a pro-inflammatory role in diseases is critical to NA treatment.

Recent studies have improved the recognition of the systems involved in NET formation, and the stimulation of pro-inflammatory factors and agents related to NETs has been proposed as a hotspot for targeted therapies for chronic inflammatory diseases in recent years. However, the mechanisms that influence and drive disease progression have not yet been clarified. Therefore, in this paper, we analyze the mechanisms of neutrophil recruitment and NET formation and their impact on the development of NA. We also discuss their possible therapeutic implications in NA, summarizing the advances in related agents and providing strategies for the treatment of NA.

## 2. Mechanisms of neutrophil recruitment

**Figure 1.**
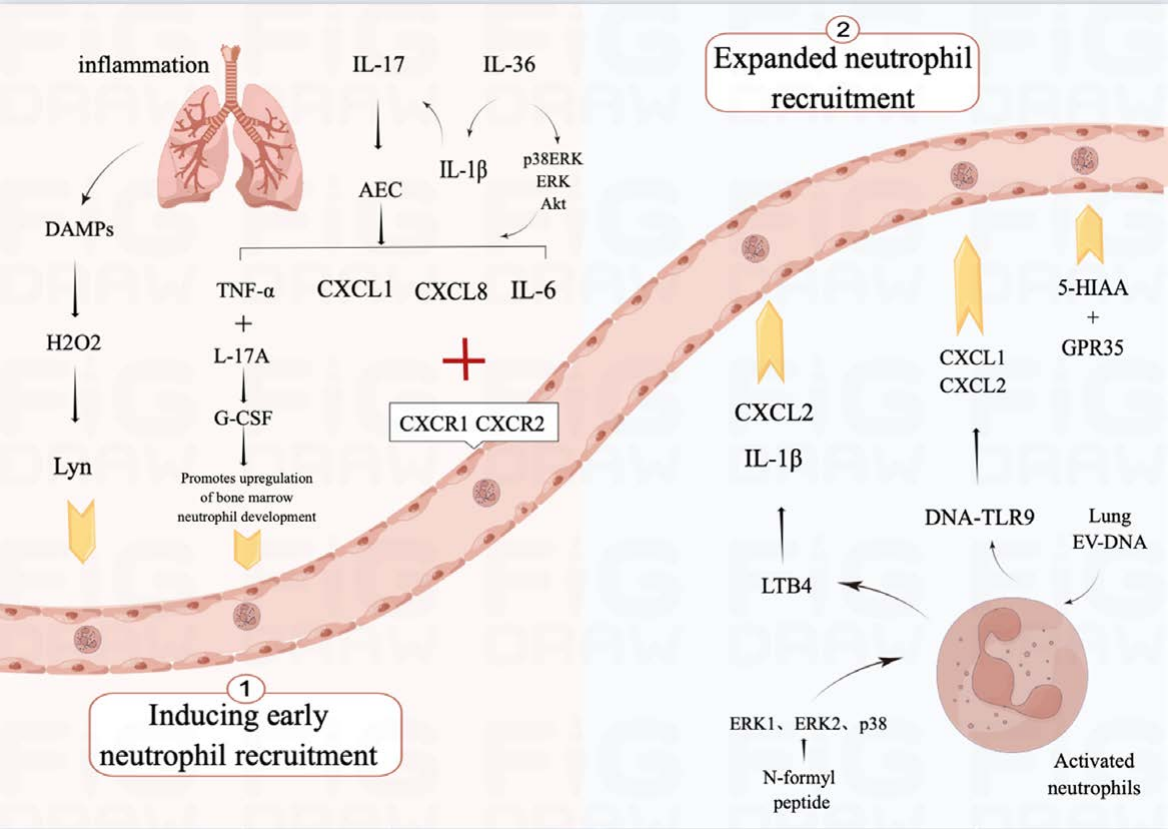
Mechanisms of neutrophil recruitment.

Neutrophils, also known as polymorphonuclear leukocytes, differentiate within the bone marrow, are the first line of defence against infection, and maintain tissue homeostasis.^[12]^ The orderly passage of mature neutrophils through blood vessels to a site of tissue inflammation or infection is known as neutrophil recruitment (Fig. 1).^[13]^ Receptors on neutrophils interact with ligands upregulated on the surface of activated (i.e., inflamed) endothelial cells, resident immune cells, and injured epithelial cells. Receptor binding initiates intracellular signaling pathways in neutrophils, inducing signaling cascades through the perception of pro-inflammatory signals (e.g., bacterial components, fungal β-glucans, or cytokines) that regulate neutrophil migration. Neutrophils are fully activated upon arrival at the site of injury, allowing for rapid and effective elimination of pathogens.^[14]^

The inflammatory response can be initiated by bacteria triggering a neutrophilic reaction or by the release of damage-associated molecular patterns (DAMPs) caused by an autoimmune response or tissue injury. DAMPs may be the earliest recruitment signals released by tissues or necrotic cells after injury and include mitochondrial DNA, *N*-formyl peptides, proteins, extracellular matrix components, and extracellular Adenosine triphosphate (ATP).^[15]^ DAMPs promote hydrogen peroxide (H_2_O_2_) release and indirect recruitment of early neutrophils via the Src family kinase Lyn acting as a redox sensor to detect H_2_O_2_ gradients. DAMPs can also directly activate the receptors on neutrophils to induce neutrophil recruitment.^[16]^ Neutrophil surface receptors respond to a variety of chemokines, which are further activated by G protein-coupled receptor-mediated signaling cascades, including the C-X-C motif (ELR-CXC chemokine), which promotes neutrophil migration.^[17]^ C-X-C motif chemokine ligand (CXCL) 8 is a potent neutrophil chemoattractant. It binds to the G protein-coupled receptors CXCR1 and CXCR2, attracting neutrophils in a concentration gradient and directing them to the site of inflammation for further antimicrobial responses.^[18]^ It is important to consider that the mechanisms of neutrophil recruitment to endothelial versus epithelial cells also differ. Epithelial cells tend to be larger than endothelial cells, and neutrophils must traverse larger distances to migrate through the former cells (≥20 µm for epithelial cells vs. 2–4 µm for endothelial cells).^[19]^ Even in cell culture models, transepithelial migration usually results in increased epithelial barrier permeability associated with more pronounced neutrophil activation and epithelial cell damage. Thus, neutrophil migration may have a negative impact on asthma because of the pathological features of increased airway epithelial permeability and epithelial cell damage in asthma. Transepithelial migration of mouse lung neutrophils was reported to depend on triggering a receptor expressed on myeloid cells (TREM)-1/3.^[20]^ TREM-1/3 deficiency resulted in reduced neutrophil counts in the airways of mice with pneumonia, which was not the case with transendothelial migration. Most means of controlling inflammation are biased towards regulating cytokine signaling, and inhibiting some migratory pathways may be a novel concept. However, partially regulating migration to ensure that enough neutrophils clear the infection is also important, and the mechanism of action of TREM-1/3, considering the specific adhesion factors required for migration through epithelial but not endothelial cells, needs to be further investigated.

Early arriving neutrophils are fully activated and can release a variety of mediators, including several granule proteins, such as NE, MPO, proteinase-3, pentraxin 3, lipid mediators (leukotriene B4, LTB4), and ROS.^[21]^ Among these, LTB4 is also a signal that induces neutrophil recruitment and promotes the neutrophil production of chemokines such as CXCL2 and the cytokine IL-1β to further amplify neutrophil recruitment. LTB4 can also be produced through the activation of the extracellular signal-regulated kinase (ERK) 1, ERK2, and p38 mitogen-activated protein kinase signaling pathways by *N*-formyl peptides. This suggests that activated neutrophils can directly and indirectly promote further secretion of CXCL8 and LTB4, increasing neutrophil recruitment. It has been demonstrated that FLAP inhibitors, such as GSK2190915, can inhibit LTB4 formation by inhibiting calcium ionophores in vitro,^[22]^ which may be important in treating NA. However, experiments have also shown no significant short-term effect on sputum neutrophil counts in asthma despite GSK2190915 inhibiting the target mediator LTB4. This suggests that inhibition of LTB4 formation alone does not fully control airway neutrophils in moderate or severe NA and that combination therapy with other agents may be required. Currently, GSK2190915 has been terminated in clinical phase II, and no ongoing clinical trials exist. In infection, other signals can also promote and amplify neutrophil infiltration, such as platelet- and mast cell-derived 5-HIAA through ligands that bind to the GPR35 receptor, which can recruit neutrophils.^[23]^ Furthermore, lung extracellular vesicles are mainly derived from type II alveolar epithelial cells, and under inflammatory conditions, by stimulating neutrophils to release chemokines CXCL1 and CXCL2 via DNA-TLR9 signaling, lung extracellular vesicle-DNA could enhance neutrophil recruitment.^[24]^

## 3. Effects of neutrophil recruitment in NA and endogenous induction factors

### 3.1. Over-infiltration of neutrophils leads to NA

Several studies have illustrated that neutrophils play a key role in the therapy of chronic inflammatory diseases such as malignancies,^[25]^ autoimmune diseases,^[26,27]^ and respiratory disorders.^[28,29]^ However, the constant recruitment and migration of neutrophils may lead to over-activation and infiltration of neutrophils at the site of tissue injury, resulting in chronic inflammation, which is detrimental to repairing damaged tissue. Some clinical forms of asthma are characterized by high airway neutrophil counts. This type of asthma, characterized by considerable infiltration in the airways and high blood neutrophil counts, is defined as NA. Although there is still no consensus on the percentage of sputum neutrophils required for a diagnosis of NA, a finding of >60% on at least 2 occasions or a neutrophil count of ≥5 to 109/L can be broadly accepted.^[30]^ Neutrophilia in the airways is uncommon in clinically stable mild asthma, but patients with chronic severe asthma can also experience neutrophilia in the non-exacerbation period.^[31]^ Patients with severe asthma or those in the exacerbation phase of asthma have more neutrophils in their airway secretions than patients with mild asthma do.^[32]^ Bronchial neutrophils are reported to be present in 54% of patients with mild to severe asthma, increasing to 68% in patients with severe asthma.^[33]^ The causes of exacerbation are mainly related to intrinsic factors associated with patient demographics (including age-related changes in neutrophil function) and co-morbidities, including obesity, body mass index, and insulin resistance, or the consequences of severe eosinophilic asthma treated with steroids.^[34]^ Steroids inhibit neutrophil apoptosis while promoting eosinophil apoptosis, leading to an increase in airway neutrophils. However, whether they are involved in recruiting neutrophils to the bronchi remains to be clarified. Although neutrophilia can co-exist with eosinophilia in some complicated cases of severe asthma, only neutrophils can be detected in sudden-onset fatal asthma.^[33]^ Neutrophil recruitment appears to be time dependent, with circulating leukocytes culminating in their migratory behavior at night,^[35]^ which may be associated with asthma exacerbations.

The above evidence indicates that excessive recruitment of neutrophils may play an essential part in the pathophysiology of NA, including severe asthma. Signalling cascades that inhibit neutrophil recruitment may be a potential means of treating NA, e.g., macrolides attenuate neutrophil inflammation-mediated airway damage by inhibiting neutrophil recruitment and activation.^[36]^ In addition, this airway neutrophilia may reflect the airway response to viral infection, which is a common trigger for asthma exacerbations. The mechanism of airway neutrophilia in severe asthma is unknown. The complexity of the role of neutrophils in disease compels us to understand the mechanisms of neutrophil recruitment and over-recruitment to improve treatments. We next discuss the endogenous inducers and effects of neutrophil recruitment in NA.

### 3.2. Endogenous inducers and effects of neutrophil recruitment in NA

IL-17 levels in bronchial biopsies are associated with airway neutrophil infiltration and IL-17 is an important cytokine associated with NA. IL-17 cytokines are secreted by Th17 cells and include IL-17A and IL-17F. The fact that neutrophils do not express IL-17 receptor C (IL-17RC) in response to IL-17A or IL-17F means that IL-17 cannot directly lead to neutrophil infiltration. Instead, IL-17A promotes neutrophil recruitment in the airway by stimulating airway epithelial cells to secrete neutrophil chemokines such as CXCL1 and CXCL8, and it induces neutrophil activation and migration via IL-6, IL-8, and tumor necrosis factor-α (TNF-α). In addition, IL-17A could enhance the contraction, migration, and diffusion of airway smooth muscle, thus promoting AHR and airway remodeling.^[37]^ IL-17A also induces mucus production in lung epithelial cells, exacerbating asthma.^[38]^ IL-17A/F and TNF-α synergistically enhanced the production of GROα and IL-8 in human bronchial epithelial cells, promoting neutrophil migration.^[39]^ In addition, the insensitivity of NA to inhaled glucocorticoids may be related to the fact that IL-17 can lead to the upregulation of glucocorticoid receptor β induced by epithelial cells.^[40]^ Binding of Th17 cytokines to IL-17RA and IL-17RC on airway smooth muscle cells promotes airway remodeling and hyperresponsiveness. Ramakrishnan et al demonstrated in a mouse model of airway remodeling that IL-17 triggers mitochondrial dysfunction by inducing fibroblast autophagy, leading to collagen deposition, which can cause severe asthmatic airway fibrosis.^[41]^ They proposed that inhibition of autophagy attenuates airway inflammation, hyperresponsiveness, and remodeling in allergic asthmatic mice, including reducing α-smooth muscle actin immunoreactivity in the airways. In some asthma model experiments, IL-17A counteracted the anti-inflammatory effects of regulatory T cells and led to direct contraction of bronchial smooth muscle cells.^[42]^ Some of these findings led to the conclusion that antibodies against IL-17A are beneficial in reducing neutrophil recruitment, airway remodeling, and hyperresponsiveness.

IL-17A normally acts as an upstream signaling factor to stimulate endothelial cells, bronchial epithelial cells, airway smooth muscle cells, and fibroblasts, leading to the production and release of the potent neutrophil attracting factors CXCL1 and CXCL8, growth factors including granulocyte-macrophage colony-stimulating factor and granulocyte-colony-stimulating factor (G-CSF), and pro-inflammatory cytokines such as TNF.^[43]^ These substances provide a suitable microenvironment for neutrophil infiltration and promote neutrophil activation and recruitment.^[44]^ Among them, G-CSF is an important downstream growth factor associated with NA.^[21]^ Recently, several reports have claimed that sputum levels of G-CSF are elevated in patients with asthma, suggesting that G-CSF is associated with the development and exacerbation of neutrophilic inflammatory asthma.^[45]^ Other reports support this view.^[46]^ For example, sputum data from patients with severe asthma showed that the expression levels of CSF3 and CSF3R were positively correlated with the severity of NA, and that neutralization of G-CSF receptors attenuated NA and mucus secretion in a mouse model.^[45]^ Kim et al^[47]^ also reported that TNF-α stimulates lung epithelial cells synergistically with factor L-17A, which promotes the expression of G-CSF and an increase in airway neutrophils by recruitment of bone marrow neutrophils. Kwak et al^[48]^ investigated the mediating role of leukotriene receptor b4 receptor 2 (BLT2) in the production of G-CSF in steroid-insensitive neutrophilic airway inflammation. They found that blockade of BLT2 inhibited G-CSF production, thereby alleviating neutrophilic inflammation in a mouse model. This suggests that the 12-LO-BLT2 cascade is also critical for G-CSF production and may serve as a target for inhibiting the progression of neutrophilic airway inflammation.

IL-36 belongs to the IL-1 family of cytokines and, together with granulocyte-macrophage colony-stimulating factor, promotes the activation of neutrophils, macrophages, and fibroblasts that exacerbate lung diseases characterized by neutrophilic inflammation, including NA.^[49]^ IL-36R has been found on human bronchial epithelial cells and lung fibroblasts, which recruit neutrophils through activation of the p38ERK, ERK, and Akt signaling pathways that regulate the expression of proteins and genes, including IL-6 and CXCL8.^[50,51]^ Luteolin has been reported to block inflammation by inhibiting the mitogen-activated protein kinase pathway mediated by IL-36γ.^[52]^ IL-36 increases the secretion of IL-1β, a cytokine that can exacerbate neutrophil recruitment by potentiating Th17-type responses, and plays an important role in severe steroid-resistant asthma.^[53]^ In addition, viruses and bacteria can amplify neutrophilic inflammation in tissues by producing IL-36 agonists. Serum amyloid A (SAA) 1 is an endogenous risk for inducing neutrophilic airway inflammation and can be triggered by viral infections.^[54]^ SAA1 is an acute-phase SAA protein that acts as an immune mediator to induce the production of pro-inflammatory cytokines and chemokines (IL-1β, IL-6, TNF-α, TGF-β1, and IL-8) by M0 macrophages and stimulate the transfer of M0 macrophages to the M1 (iNOS, CD86) phenotype. This promotes sustained neutrophilic airway inflammation by shifting the differentiation process of M1 macrophages and TGF-β1-mediated airway remodeling.^[55]^ In addition, SAA1 stimulates AECs, neutrophil activation, and NET formation.^[55]^ Therefore, NA combined with viral and bacterial infections is important as it may cause NA exacerbation.

### 3.3. Therapies inhibiting neutrophil recruitment

Elevated levels of CXC chemokine expression are associated with increased neutrophil infiltration in asthmatic bronchial mucosa, the airway wall, and sputum.^[56]^ The chemokine receptor CXCR2 mediates neutrophil recruitment and induces NET formation via IL-8. AZD5069 is a selective CXCR2 antagonist developed by AstraZeneca to prevent neutrophil recruitment without hampering neutrophil immune defences.^[57]^ AZD5069 significantly reduced neutrophil infiltration in sputum and bronchial biopsies in patients with severe asthma (90% inhibition).^[58]^ However, concerns about the ability of AZD5069 to treat poorly controlled severe NA also exist. This selective CXCR2 antagonist was used in a trial to treat patients with severe asthma, but it was not effective in controlling disease deterioration.^[59]^ The participants in this experiment did not smoke, as smoke exposure can lead to upregulation of CXCL8 levels in the airway mucosa, exacerbating neutrophil recruitment and NET formation. Thus, the therapeutic potential of AZD5069 for asthmatic smokers remains to be investigated. Dompé Pharmaceuticals has developed Ladarixin, an oral selective dual CXCR1 and CXCR2 antagonist currently being tested in phase II clinical trials (NCT02814838 and NCT01571895).^[60]^ Results of a trial showed that treatment with Ladarixin reduced MPO, IL-8, and NE levels, as well as neutrophil counts, but the patients’ respiratory parameters did not significantly improve.^[61]^ Another oral dual CXCR1/CXCR2 antagonist, SCH 527123, an effective and safe treatment for severe NA, has been developed by Merck Sharp & Dohme LLC for the treatment of severe NA and is currently completing phase II clinical trials.^[62]^ SCH 527123 reduces circulating and airway neutrophil levels by targeting neutrophil migration and has no toxic effect on granulocyte progenitors in the bone marrow.^[61]^

## 4. Triggers and molecular mechanisms of NETs

**Figure 2.**
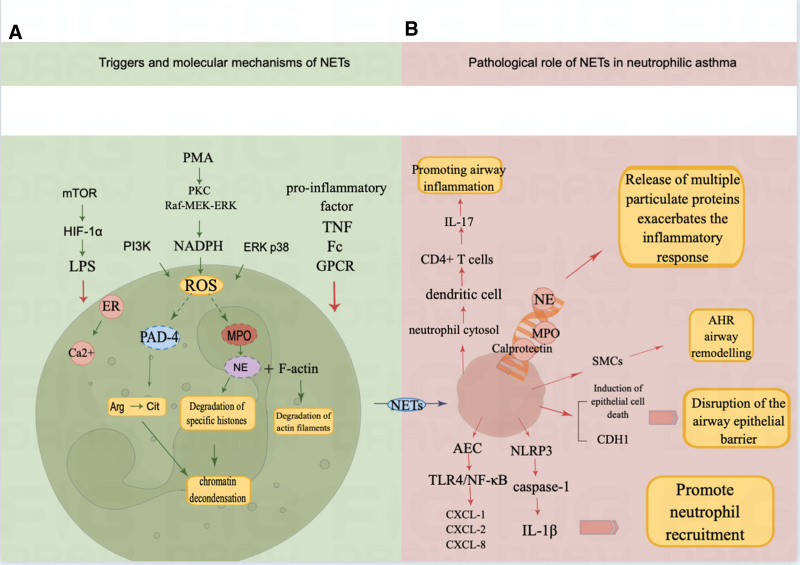
(A) Triggers and molecular mechanisms of NETs. (B) Pathological role of NETs in neutrophil asthma. NETs = neutrophil extracellular traps.

NETosis is primarily a result of neutrophil death, although the process is distinct from apoptosis and necrosis.^[63]^ NETosis starts with the activation of neutrophil surface receptors and involves 4 key steps that occur over a few hours: permeabilisation of the plasma membrane, disassembly of the cytoskeleton and nuclear membranes, uncoiling of chromatin, and assembly of antimicrobial proteins into the chromatin scaffold. Another formation mechanism is non-soluble NETosis, where NETs can undergo rapid release without cell death by neutrophils secreting chromatin and granular contents.^[64]^ This review focuses on suicidal NETosis as it is an important mediator of inflammation. The process of NETosis, from initiation to formation, and the important factors and molecular mechanisms that advance this process are described next (Fig. 2).

Triggering NETosis requires the activation of neutrophils. The most potent inducer of NETosis in vitro is phorbol 12-myristate 13-acetate (PMA), which stimulates neutrophils. In addition, ligands for lipopolysaccharide (LPS), G protein-coupled receptor,^[17]^ TNF,^[65]^ and Fc receptor^[66]^ can trigger NETosis. It has been reported that mice lacking Toll-like receptor 2 (TLR2) or complement component 3 and those with defective IL-1 receptor/TLR signaling stimulated with *Staphylococcus aureus* do not release NETs from their neutrophils.^[64]^ NETs can also be induced by stimulation with pro-inflammatory factors, such as IL-33,^[67]^ CXCL8,^[68]^ IL-17,^[69]^ and C3a/C5a,^[70]^ which are important inducers of NET formation and are associated with asthma pathophysiology. In an animal experiment,^[71]^ higher levels of inflammation, neutrophil counts, and NET were found in the airways of NA mouse models. Many novel stimuli have been found to induce NET formation, which damages epithelial cells and exacerbates NA. In recent years, benzyl phthalate, a widely used plasticizer, has been found to promote extensive neutrophil infiltration in bronchoalveolar lavage fluid and deposition of NETs in lung tissue.^[72]^ Mono-n-butyl phthalate, an interfering chemical, induces activation and release of NETs from human neutrophils, mediates macrophage polarization towards M1, and causes airway epithelial cell damage. However, treatment with hydroxychloroquine (an autophagy inhibitor) can reduce the symptoms.^[73]^

Activation of neutrophils leads to actin polymerization and increased intracellular Ca^2+^ and ROS generation. Cytoskeletal dynamics are critical in NET formation and require cysteine glutathionylation of actin and microtubule proteins, and inhibition of actin polymerization impairs NETosis.^[74]^ Cysteine glutathionylation of actin and microtubule proteins was found to be mediated by ROS, which orchestrate degradation of the actin cytoskeleton through activation of NE.^[75]^ Activation of the receptor in neutrophils triggers calcium release from endoplasmic reticulum stores, which opens plasma membrane calcium channels.^[76]^ Thus, increased intracellular calcium can be observed in LPS-, IL-8-, or PMA-stimulated neutrophils.^[77]^ PMA activates reduced nicotinamide adenine dinucleotide phosphate oxidase via PKC and Raf-MEK-ERK signaling pathways, generating ROS.^[78]^

ROS represent an important mediating hub in the NETosis process. NET formation can also be stimulated by certain NET stimulants, such as immune complexes, ionomycin, and nicotine, which are not dependent on nicotinamide adenine dinucleotide phosphate-generated ROS but rather on mitochondrial-generated ROS.^[79]^ Non-soluble NETosis is also thought to occur independently of ROS.^[80]^ ROS can also be induced by phosphatidylinositol 3-kinase (PI3K),^[81]^ ERK, and p38.^[82]^ Selective PI3K inhibitors have been developed as novel treatments for steroid-resistant chronic inflammatory airway diseases. These inhibitors reverse corticosteroid insensitivity by increasing HDAC2 activity and improve airway inflammation by modulating endoplasmic reticulum stress.^[83,84]^ Dual PI3Kδ/γ inhibitors such as TG100-115 and IPI-145 are being evaluated in clinical trials for the treatment of asthma and chronic obstructive pulmonary disease (COPD), showing positive results.^[85,86]^ In a study of asthmatic mice,^[87]^ toluene diisocyanate produced elevated NET levels induced through the p38 and ERK signaling pathways, promoting elevated IL-8 levels. A small molecule drug, necro inhibin-1, was found to inhibit the formation of NETs by modulating the ERK pathway, reducing total protein, MPO, and inflammatory cytokine levels in the bronchoalveolar lavage fluid to alleviate neutrophil-dominated airway inflammation.^[88]^ The p38 inhibitor PH-797804 has completed phase II clinical trials in moderate to severe COPD without significantly improving clinical outcomes.^[89]^ The p38 inhibitor AZD7624 showed no benefit in treating patients with COPD with upregulated p38α levels.^[90]^ A search of ClinicalTrials.gov indicated that AZD7624 is undergoing a phase II clinical trial for the treatment of corticosteroid-resistant asthma, but no results were available. Thus, targeting a particular upstream signal is not sufficient to inhibit severe neutrophil infiltration, and further searches for suitable target signals are required. In addition, ROS are involved in the activating effects of downstream signaling. ROS stimulate MPO, which, when activated, can be released from eosinophilic granules and produce highly effective antimicrobial activity with the aid of H_2_O_2_.^[91]^ Triggered by activated MPO, the concentration of NE in the cytoplasm gradually increases as it decreases from the MPO-containing nitrogen plasma complex. At the same time, actin filaments are degraded via the binding of NE and F-actin in the cytoplasm.^[92]^ In addition, MPO binds to chromatin and, together with NE, depolymerizes chromatin independently of its enzymatic activity, prompting the release of DNA into the extracellular space to capture invading pathogens.^[93]^ Not only does ROS-activated MPO help to depolymerize chromatin, but the chlorinated polyamines generated by the reaction of hypochlorite produced by MPO also cross-link with the NET proteins, thereby improving the stability and integrity of the NETs and enhancing their ability to capture microorganisms.^[94]^ This cross-linking may be the reason that NET proteins survive longer than DNA after administration of deoxyribonuclease I (DNase I) in vivo. In addition, DEK, another nuclear-chromatin-binding protein that promotes chromatin depolymerization similar to that of MPO, serves as a potential target for the treatment of joint inflammation.^[95]^

ROS-activated peptidyl arginine deiminase (PAD)-4 is also an important controller of NETosis. Histones are the structural units of nucleosomes responsible for the compressing and loading of DNA into chromosomes, and the citrullination of histones, also known as deimination, is the conversion of arginine to citrulline residues catalyzed by the PAD family.^[96]^ PAD4 catalyzes histone hypercitrullination, which can reduce the positive charge of histones and their interaction with DNA, affecting chromosome structure, and leading to a reduction in hydrogen bonding and chromatin depolymerization, a key step in the formation of NETs.^[97]^ This process requires the binding of 5 calcium ions,^[98]^ which are provided by the activation of neutrophils and subsequent increases in intracellular Ca^2+^. PAD-4-deficient mouse neutrophils fail to release NETs in response to LPS and TNF stimulation.^[99]^ Simvastatin inhibits NETosis neutrophilic inflammation and AHR by reducing PAD-4 expression in a mouse model of severe asthma.^[100]^ In addition, H3cit has been suggested as a possible novel and specific biomarker for this process.^[101]^ The clinical relevance of increased serum H3cit to the mechanism of asthma progression and risk of exacerbation remains to be determined, and no effective biological agents are available.

Some evidence suggests that autophagy might be involved in the formation of NETs. Rapamycin regulates the autophagic response and, when used as a mammalian target, mediates LPS-triggered NET formation by transcriptionally controlling the expression of hypoxia-inducible factor 1’s alpha subunit.^[80]^ PI3K not only induces ROS but is also an important kinase required for the induction of autophagy.^[81]^ Premyeloid cells lacking the autophagy-related protein ATG7 exhibit a modest reduction in NET release.^[102]^ The conversion of LC3B-I to LC3B-II is an important hallmark of autophagy activation and is found during NET formation.^[103]^ In neutrophils stimulated with PMA and LPS, autophagosomes containing cytoplasmic components were observed by transmission electron microscopy.^[104]^ Hydroxychloroquine, an autophagy inhibitor, treats AEC damage caused by the mono-n-butyl phthalate-induced activation of human neutrophils and release of NETs.^[73]^

## 5. Pathological role of NETs in neutrophilic asthma

In neutrophilic asthma, the formation of NETs is abnormally increased, leading to airway inflammation and airway remodeling. Exploring the concentration of NETs in the circulatory system and alveoli is of great significance for understanding the pathogenesis of neutrophilic asthma and finding new therapeutic targets.

Circulating concentration of NETs: NETs can reach the lungs through the blood circulation and participate in airway inflammation and airway remodeling. The concentration of NETs in the blood of patients with neutrophilic asthma increases, which is related to the severity of the disease and airway inflammation indicators.

Alveolar concentration of NETs: NETs can form in the alveoli, causing alveolar inflammation and damage. The concentration of NETs in the bronchoalveolar lavage fluid of patients with neutrophilic asthma increases, which is related to airway inflammation and airway remodeling indicators.

### 5.1. NETs promote neutrophil recruitment

C-X-C motif chemokines (ELR-CXC chemokines) are important promoters of neutrophil recruitment.^[18]^ In asthma, NETs exacerbate neutrophil recruitment and inflammation by stimulating airway epithelial cells to activate the TLR4/NF-κB pathway and secrete CXCL-1, CXCL-2, and CXCL-8 chemokines.^[71]^ IL-8 (CXCL8), a potent NET inducer, is elevated in NA and can further circulate, exacerbating neutrophil accumulation.^[105,106]^ Furthermore, activation of the nucleotide-binding oligomeric structural domain-like receptor family pyridine structural domain containing 3 (NLRP3) inflammasome plays a critical role in the development of NA.^[107,108]^ NETs can activate the NLRP3 inflammasome to trigger caspase-1 to release IL-1β,^[109]^ whose release promotes neutrophil recruitment to the lungs, further perpetuating the cycle of inflammation and leading to excessive inflammation and tissue damage.^[110]^ In addition to the previously described CXCR2 antagonists, a multifunctional nanotherapeutic, LaCD NP, has been proposed in a recent study as a multifaceted treatment for NA through inhibition of neutrophil migration/infiltration as well as attenuation of NET formation and activation of the NLRP3 inflammasome in neutrophils.^[111]^ Moreover, LaCD NP inhibits macrophage-induced inflammation and attenuates airway epithelial cell death and smooth muscle cell proliferation by alleviating neutrophilic inflammation. However, many cellular and molecular mediators are involved in the development and progression of asthma, and there are limitations in the number of specific molecules whose anti-asthmatic activity can be targeted by nanotherapies. Therefore, although the study results hold promise for the treatment of NA with LaCD, the efficacy against NA needs to be further elucidated.

### 5.2. NETs affect airway epithelial cells and pro-inflammatory factors

TNF, which triggers NET formation, is divided into 2 types – TNF-α originating from monocytic phagocytes and TNF-β originating from T lymphocytes. IL-17 is the main stimulator of TNF. TNF-α is elevated in NA and severe asthma and is strongly correlated with lung function, asthma control, and age; treatment with azithromycin also effectively reduces inflammation.^[112]^ Moreover, TNF-α induces the effects of NETs on NA and enhances airway smooth muscle contraction, possibly contributing to AHR.^[113]^ Biological therapies such as anti-IL-17R and anti-TNF-α can effectively treat NA by interfering with pro-inflammatory signaling pathways.^[114]^ Beneficial effects of the soluble TNF-α receptor blocker etanercept on clinical outcomes have been reported in clinical studies,^[115,116]^ but the results in controlled trials have not been supportive.^[117]^ Another TNF-alpha receptor blocker, golimumab, has shown high levels of malignancy rates and infections in clinical trials and is therefore considered unsuitable for further investigation in the treatment of asthma.^[118]^

Neutrophil cytosol is detected in severe NA as a denucleated cytosol released during NETosis. Cytoplasmic bodies participate in airway inflammation by activating lung dendritic cells in vitro and triggering CD4 + T cells to produce antigen-specific IL-17.^[119]^ High levels of NETs were produced in a neutrophilic animal model of asthma and correlated with the severity of airway inflammation, and inhibition of NET production reduced airway inflammation and hyperresponsiveness.^[120]^ In addition, NETs may stimulate the proliferation of airway smooth muscle cells and contribute to AHR and airway remodeling.^[121]^ NETs induce epithelial cell death and regulate E-calmodulin expression, disrupting epithelial barrier integrity, a process that α-1 antitrypsin has been found to limit.^[122]^ In a study, NETs induced upregulation of IL-6 and IL-8 mRNA in AEC, exacerbating airway inflammation.^[108]^ It was also reported that NET stimulation significantly upregulated the expression of α-smooth muscle actin, Twist, and Snail proteins, suggesting that NETosis promotes epithelial-mesenchymal transition in lung epithelial cells, leading to epithelial cell damage, which can be interfered from and reversed by the JAK2 inhibitor tofacitinib.^[123]^ Furthermore, NETs have been shown to enhance asthma severity by damaging airway epithelium and triggering an inflammatory response in alveolar epithelial cells.^[124]^ However, circulating NET concentrations appear to be more representative of asthma severity than alveolar NET concentrations.^[125]^ Therefore, the study of NETs has not yet been standardized and further exploration is needed.

### 5.3. NETs release multiple particulate proteins that exacerbate airway inflammation

NETs release extracellular structures containing DNA and several granule proteins, such as NE, MPO, and calcineurin,^[29]^ which are used to trap and kill pathogens and induce the secretion of pro-inflammatory cytokines associated with airway neutrophils and airway inflammasomes.^[126]^

NE and MPO are 2 major granule proteins in NETs associated with airway epithelial and cellular damage.^[127]^ Blocking exposure to these 2 particulate proteins by pre-incubation with antibodies against NE or MPO, as well as enzymatic degradation of histones with proteinase K, reduces the toxicity of NETs and attenuates airway epithelial damage.^[128]^ In addition, as mentioned previously, NE can be activated by MPO to translocate to the nucleus and trigger the release of NETs, and it can also stimulate the production of a variety of inflammatory chemokines such as IL-8.^[129]^ Therefore, inhibition of NE activity not only inhibits NET formation, but also reduces inflammation, which reduces tissue damage.^[130]^ GW311616A is an NE-selective inhibitor that prevents NETosis by blocking NE nuclear translocation and subsequent chromatin decondensation.^[28]^ Mice treated with GW311616A in bronchial alveolar lavage fluid showed a significant reduction in the production of NETs in the lungs, with significant attenuation in the number and percentage of neutrophils as well as in the levels of neutrophil chemotactic factors (LTB4, KC, and CXCL5) and pro-inflammatory cytokines (IL-1β and TNF-α). Whether this has the potential to be used as a treatment for NA is not certain, and clinical trials are needed.

Calprotectin, a heterodimeric protein complex released by NETs, consists of S100A8 and S100A9 subunits. In one study, the serum levels of calprotectin were elevated in patients with asthma and correlated with lung function, smoking, and blood neutrophil ratios in patients with asthma.^[131]^ It was found that peripheral blood neutrophils from patients with asthma induced airway epithelial cells to produce S100A9, which further activated AECs through the ERK pathway, stimulated NET formation, and induced M1 macrophage polarization.^[132]^

Elevated sputum extracellular DNA (eDNA) levels in patients with severe asthma correlated positively with sputum neutrophils, correlated negatively with predicted FEV1%, and resulted in elevated CXCL-8, IL-1β, and caspase-1 activities.^[108]^ Rhinovirus-induced NETs release elastase and dsDNA, producing more inflammatory factors such as IL-1β, leading to worsening acute asthma.^[133]^ Therefore, sputum eDNA can be associated with increased neutrophil numbers and activation in individuals with asthma, and targeting dsDNA release has been suggested as a potential therapeutic strategy to alleviate NA.^[134]^ DNase I is capable of degrading ssDNA and dsDNA to release 5′-phosphate and 3′-hydroxyl oligonucleotides, and can be used to remove DNA from protein samples, thereby disrupting NETs and effectively inhibiting AHR and inflammation.^[135]^ NETs have been reported to cause glucose-6-phosphate dehydrogenase release and subsequent AEC injury, and disruption of NETs by DNase prevents this process. Inhaled DNase can be speculated to prevent NET-mediated airway epithelial cell injury and have an outstanding therapeutic effect in patients with high eDNA levels.^[108]^ DNase I has been shown to cleave the phosphoanhydride bond preferentially but it can also degrade ATP and ADP, which are important agonists for neutrophil activation.^[136]^ Thus, DNase I has also been shown to inhibit neutrophil activation by degrading ATP and ADP.^[137]^ In an experiment, DNase I could regulate IL-8 and MKP1 mRNA expression by inhibiting NETs and ameliorating toluene diisocyanate-induced airway epithelial barrier disruption in asthmatic mice.^[86]^ However, the low serum stability of DNase I is a limitation of its use in clinical applications. To break this limitation, the binding of DNase I to a microgel (DNase I MG) synthesized from highly hydrophilic N-(2-hydroxypropyl) methacrylamide and zwitterionic carboxybetaine methacrylamide has been investigated, which resulted in increased enzyme bioavailability.^[138]^ The protein repulsive nature of DNase I MGs allows them to degrade NETs more efficiently than free DNase I in biological media, even after prolonged exposure to neutrophils that are constantly releasing NETs.^[138]^

### 5.4. NET release is physiologically regulated

It was mentioned earlier that neutrophil recruitment may be time dependent. The transcription factor Bmal1 (circadian cycle regulator) has been shown to control neutrophil degranulation and NET formation through CXCL2-CXCR2 signaling.^[139]^ Although the number of neutrophils versus platelets does not affect NET formation, the quantity of granules in neutrophils is an important factor in the formation of NETs. Neutrophils are mobilized into the bloodstream at night and lose the capacity to form NETs during the day with the loss of granules.^[138]^ This circadian regulation is also correlated with the severity of lung inflammation; significantly less lung damage from inflammation can be observed in mutant mice with a low daytime granule content.^[139]^ Thus, the circadian rhythm in neutrophil responses helps us predict NET formation and intervene accordingly.

Age is also associated with the formation of NETs. A study indicated that older adult humans and mice (averaging approximately 70 years and 18 months, respectively) have a reduced response to neutrophil stimulation and a markedly reduced NET formation capacity,^[140]^ possibly related to reduced ROS production or defective autophagy.^[141]^ We must, therefore, consider age in our studies, whether granules released from NETs affect inflammation differently in different age groups, and how they should be addressed in therapy.

## 6. Summary and outlook

Asthma is a complex heterogeneous disease, and the high morbidity, mortality, and economic costs of severe asthma remain a major global public health problem that needs to be urgently addressed due to the enormous social and personal burdens worldwide. Steroids are usually effective in the treatment of asthma; however, their efficacy in neutropenic asthma is weak. The mechanism of neutrophil involvement in the pathogenesis of severe asthma has not been clearly elucidated, but current research supports a strong association between severe asthma and excessive neutrophil infiltration and higher levels of NETs. The search for potential therapeutic targets to develop steroid substitutes is the focus of current research (Table 1).

**Table 1:** Preparations for the treatment of NA.

Objectives	Preparations	Clinical trial	References
CXCR1	Ladarixin	Phase II	^[60,61]^
CXCR2	SCH 527123	Phase II	^[61,62]^
CXCR2	AZD5069	Phase II	^[57–59]^
LTB4	GSK2190915	Terminated	^[22]^
PI3	TG100-115	Phase II	^[84]^
	IPI-145	Phase II	^[85]^
P38	AZD7624	Phase II (COPD)	^[89]^
	PH-797804	Phase II (COPD)	^[88]^
ERK	Nec-1	Not available	^[87]^
TNF-α	Etanercept	Inapplicable	^[114–116]^
	Golimumab	Terminated	^[117]^
NLRP3	LaCD NP	Not available	^[110]^
NE	GW311616A	Not available	^[130]^
dsDNA	DNase I	Phase II	^[86,132,134–136]^
	DNase I MGs	Not available	^[137]^

COPD = chronic obstructive pulmonary disease, DNase I = DNA after administration of deoxyribonuclease I, DNase I MG = the binding of DNase I to a microgel, NA = neutrophilic asthma.

Neutrophilic asthma is a special type of asthma with a different pathogenesis from other types of asthma. By demonstrating the role of neutrophils and NETs in neutrophilic asthma, it can not only help readers better understand the pathogenesis of this subtype of asthma, provide new ideas for the prevention and treatment of asthma, but also discover new therapeutic targets for neutrophilic asthma, provide a theoretical basis for the development of new therapeutic drugs, thereby improving the level of diagnosis and treatment, and promoting the research progress in the field of asthma.

The therapeutic strategies for NA mentioned in this article are broadly categorized into 2p types. One strategy blocks the trigger signals for neutrophil activation and NET formation, and the other inhibits neutrophils and the granules released from NETs. Migration of neutrophils is mainly controlled through the activation of CXCR1 and CXCR2 receptors by CXC chemokines. Neutrophil migration can be directly inhibited by blocking CXC chemokines. CXCR2 blockers are novel small-molecule inhibitors that have been explored for the treatment of neutrophil-related diseases. However, the clinical efficacy of AZD5069 is controversial. In recent years, newer dual CXCR1 and CXCR2 blockers such as Ladarixin SCH 527123 have also entered phase II clinical trials and show great therapeutic potential in safety and efficacy. In addition to directly blocking neutrophil recruitment, signals that enhance neutrophil recruitment, such as LTB4, can be targeted, but the clinical effect is not obvious and may require combination with other drugs. NET formation can be triggered by several signals, such as the PI3K, p38, and ERK signaling pathways, and by TNF. Dual PI3Kδ/γ inhibitors such as TG100-115 and IPI-145P38 positively impact clinical outcomes. However, p38 and ERK signaling pathways have only been shown to effectively inhibit NETs in basic research studies, and there are no active pharmacological agents currently in clinical trials. A small molecule drug, necro inhibin-1, has been found to modulate the ERK signaling pathway to inhibit NETs, which is promising as a drug for the treatment of NA. Etanercept, a soluble TNF-α receptor blocker, showed significantly positive outcomes in initial clinical trials, but objections were raised in larger controlled trials. Another TNF-α receptor blocker, golimumab, was discontinued from trials after questions arose about its safety. LaCD NP is a multifunctional nanotherapeutic that blocks both neutrophil recruitment and NETs and inhibits NLRP3 inflammasome activation. However, LaCD NP still has limitations, and its efficacy against NA remains to be further elucidated, as proposed in a recent study. NE, MPO, and calprotectin are released during NET formation. NE-selective inhibitors such as GW311616A resulted in a significant reduction in the production of NETs in the lungs, with significant attenuation in the number and percentage of neutrophils as well as in the levels of neutrophil chemotactic and pro-inflammatory cytokines. However, its potential in the treatment of NA has yet to be evaluated in clinical trials. DNase I can disrupt NETs by degrading ssDNA and dsDNA and has been used as an inhibitor of NETs in several studies. Modified DNase I MGs increase the serum stability of DNase I and improve its efficiency to degrade NETs. In addition, biomarkers for NETs continue to emerge that have been proposed as therapeutic targets for NA, such as H3cit, G-CSF, and S100A9, but all lack standardization and clinical support. Since NETs may be associated with autophagy, inhibitors of autophagy (e.g., hydroxychloroquine) might also have a therapeutic effect on NA.

The above twists and turns in the study of drugs for the treatment of NA reflect the complexity of the NA mechanism. Inhibiting one conduction signal alone is not sufficient to significantly decrease neutrophil levels at the lesion site, thus requiring further in-depth exploration and experimentation on the mechanisms involved in NA. The influence of other factors such as smoking, circadian rhythms, and age must also be considered in our research and may impact treatments.

## Acknowledgments

Over the course of my researching and writing this paper, I would like to express my thanks to all those who have helped me. First, I would like to express my gratitude to all those who helped me during the writing of this thesis. A special Acknowledgments should be shown to Professor Baihua Jiang, from whose lectures I benefited greatly. I am particularly indebted to Mr. Yang who gave me kind encouragement and useful instruction all through my writing. And my warm gratitude also goes to my friends and family who gave me much encouragement. Moreover, I wish to extend my thanks to the library and the electronic reading room for providing much useful information for my thesis.

## Author contributions

**Conceptualization:** Baihua Jiang.

**Writing – original draft:** Yuemu Li.

**Writing – review & editing:** Tianyi Yang.
